# Monocyte Response to Different *Campylobacter jejuni* Lysates Involves Endoplasmic Reticulum Stress and the Lysosomal–Mitochondrial Axis: When Cell Death Is Better Than Cell Survival

**DOI:** 10.3390/toxins10060239

**Published:** 2018-06-13

**Authors:** Barbara Canonico, Gianna Di Sario, Erica Cesarini, Raffaella Campana, Francesca Luchetti, Loris Zamai, Claudio Ortolani, Maria Gemma Nasoni, Wally Baffone, Stefano Papa

**Affiliations:** Department of Biomolecular Sciences, University of Urbino Carlo Bo, 61029 Urbino, Italy; gianna.disario@uniurb.it (G.D.S.); erica.cesarini@uniurb.it (E.C.); raffaella.campana@uniurb.it (R.C.); francesca.luchetti@uniurb.it (F.L.); loris.zamai@uniurb.it (L.Z.); claudio.ortolani@uniurb.it (C.O.); maria.nasoni@uniurb.it (M.G.N.); wally.baffone@uniurb.it (W.B.); stefano.papa@uniurb.it (S.P.)

**Keywords:** *Campylobacter jejuni*, apoptosis, sub-lethal effect, lysosome, mitochondria

## Abstract

*Campylobacter jejuni* is a Gram-negative spiral-shaped bacterium, commonly associated with gastroenteritis in humans. It explicates its virulence also by the cytolethal distending toxin (CDT), able to cause irreversible cell cycle arrest. Infection by *C. jejuni* may result in the development of the Guillain–Barré Syndrome, an acute peripheral neuropathy. Symptoms of this disease could be caused by CDT-induced cell death and a subsequent inflammatory response. We tested *C. jejuni* lysates from different strains on donor monocytes: in fact, monocytes are potent producers of both pro- and anti-inflammatory cytokines, playing a major role in innate immunity and in non-specific host responses. We found, by cytometric and confocal analyses, that mitochondria and lysosomes were differently targeted: The *C. jejuni* strain that induced the most relevant mitochondrial alterations was the ATCC 33291, confirming an intrinsic apoptotic pathway, whereas the *C. jejuni* ISS 1 wild-type strain mostly induced lysosomal alterations. Lysates from all strains induced endoplasmic reticulum (ER) stress in monocytes, suggesting that ER stress was not associated with CDT but to other *C. jejuni* virulence factors. The ER data were consistent with an increase in cytosolic Ca^2+^ content induced by the lysates. On the contrary, the changes in lysosomal acidic compartments and p53 expression (occurring together from time 0, T0, to 24 h) were mainly due to CDT. The loss of p53 may prevent or impede cell death and it was not observable with the mutant strain. CDT not only was responsible for specific death effects but also seemed to promote an apoptotic stimuli-resisting pathway.

## 1. Introduction

*C. jejuni* is a gram-negative spiral-shaped bacterium, commonly associated to gastroenteritis in humans. It can penetrate and damage the intestinal mucosa, leading to blood and inflammatory cells in stool [1]. *C. jejuni* infection is related to both mild diarrhea and severe inflammatory enteritis, although the mechanisms of pathogenesis are still poorly understood. *C. jejuni* infection is a multistep process that includes interaction with and invasion of the intestinal epithelial cells (IECs) [2]. It is reported that *C. jejuni* can invade human IECs either via paracellular or transcellular routes [3,4]. *C. jejuni* seems to survive intracellularly in human monocytes which, once in circulation, migrate to tissues where they can differentiate into macrophages or specific types of dendritic cells [5]. Van Rhijn I. et al. demonstrated that *Campylobacter* DNA can persist in circulating human peripheral blood mononuclear cells (PBMC) [6]. Despite an intracellular niche for *C. jejuni* survival has not been well described yet, human myelomonocytic cells are eligible candidates. *C. jejuni* infection is mostly self-limiting; nevertheless, in some cases (1/1000 to 1/2000), serious life-threatening complications can develop, such as the Guillain–Barré syndrome [7,8]. This paralyzing syndrome is an acute peripheral neuropathy causing progressive limb weakness, coupled with glove or stock-like sensory disturbance [9,10]. It is very likely that these symptoms are caused by cell death induced by the cytolethal distending toxin (CDT) and its subsequent inflammatory response [11,12]. 

To date, the identified bacterial factors implicated in host cell invasion and disease pathogenesis are lipooligosaccharides, the capsule, the flagellar apparatus, the cytolethal distending toxin (CDT), and the post-translational glycosylation system (O-linked and N-linked glycosylation).

*C. jejuni* is also able to produce outer membrane vesicles (OMVs) that contain biologically active CDT: during pathogenesis, the release of OMVs by *C. jejuni* is a route through which this bacterium delivers all CDT subunits to the surrounding environment, infecting host cells and causing the typical cytolethal distending effects.

CDT is a heterotrimeric holotoxin belonging to the subclass of the AB_2_ toxin superfamily. CDT comprise three subunits, CdtA, CdtB, and CdtC. The A subunit of the toxin, CdtB, exhibits cation-dependent metalloenzyme activities in vitro, characteristic of endonucleases [13,14], inositol polyphosphate 5-phosphatases [15], and sphingomyelinases [16]. The B component consist of two heterogeneous subunits, CdtA and CdtC, that act as carriers to deliver the catalytic subunit, CdtB, into host cells [12]. CdtB reaches the nucleus by endoplasmic reticulum(ER)-associated degradation (ERAD) or non-ERAD pathways (followed by translocation across the nuclear membrane) where it exhibits DNase I-like activity and induces limited DNA damage such as double-strand damage, leading to the activation of DNA repair responses and cell cycle arrest at the G2/M phase [14,17]. 

Apoptosis is an ordered cellular process triggered by various signaling pathways, notably, the intrinsic (or mitochondrial) and the extrinsic pathways; both pathways initiate caspases activation [18]. Apoptosis consists of a sequence of characteristic biochemical changes, such as mitochondrial outer membrane permeabilization (MOMP), activation of the effector caspases, and activation of catabolic hydrolases that degrade most of the macromolecules of the cell, including DNA [19]. Because MOMP effectively represents a point of no return, it is highly regulated, largely by members of the Bcl-2 protein family. Overexpression of Bcl-2 as well as deficiency of Bax and Bak can confer protection against lethal ER stress [20,21]. Recent findings indicate that stress signals are relayed from the ER to the mitochondria and that ER stress-induced apoptosis, similar to mitochondrial-mediated apoptosis, is also regulated by the Bcl-2 family of proteins [22,23,24]. 

In recent years, numerous bacterial pathogens, such as *Helicobacter pylori*, *Chlamydia trachomatis*, *Shigella flexneri*, and *Neisseria gonorrhoeae* have been shown to inactivate p53, through the ubiquitin-proteasome degradation system, blocking the protective response of the host cell. p53 inhibits cell growth by up- and downregulating genes involved in apoptosis, cell cycle, senescence, differentiation, DNA repair, transcription, translation, as well as cytoskeleton, cell adhesion, angiogenesis, and migration [25]. These findings demonstrate that p53 has many functions in genomic stability control, apoptosis, metabolism, and antioxidant defence [26]. Apoptosis induction is one of the main tumor suppressor activities of p53 [27]. p53 degradation seems to prolong the survival of the pathogen’s niche; nevertheless, also other ways to modulate p53 were observed. However, this evidence supports the hypothesis that p53 mediates host defense and cell fate during bacterial infection.

An association of inflammation, upregulation of p53 levels, and CD59 (MIRL) [28,29] was reported, and it was supposed that p53 is a direct regulator of the immune response by modulating CD59 levels. Furthermore, the functions of p53 and ICAM-1 (also known as CD54) are related: p53 and ICAM-1 have an intracellular guardian role, and their functions seem to be linked in various physiological and pathological settings [30,31]. ICAM-1 is a crucial receptor involved in cell–cell interactions and may either mediate or enhance the invasion of a number of pathogenic organisms; an increased ICAM-1 expression induced by various pathogens was shown to mediate cell-to-cell adhesion in inflamed tissues [31,32].

In many cell death events, lysosomes are involved. The contribution of lysosomes may be active (leading to cell death) or passive (a consequence of cell death), and can also amplify the cell death response [33]. Several lysosomal proteins have been shown to affect both autophagy and cell death. On the contrary, lysosomes may also play a key role in resealing the membrane to prevent cell death [34]. LAMP-1, a major integral membrane glycoprotein of late endosomes and lysosomes, is an activation-dependent cell surface glycoprotein in human peripheral blood mononuclear cells which mediates cell adhesion to the vascular endothelium [35].

We described how human monocytes interact with cell lysates from two CDT producer strains, namely, *C. jejuni* ATCC 33291 and *C. jejuni* ISS 1, and a mutant strain, *C. jejuni* 11168H *cdtA*. We adopted the cellular model of monocytes, accounting for 3–8% of leukocytes in the peripheral blood [36]. Known to be effective producers of both pro- and anti-inflammatory cytokines, monocytes play a major role in innate immunity and in non-specific host response against both exogenous pathogens—primarily by phagocytosis—and endogenous substances created by tissue damage [37]. CD14 is a molecule linked to Toll-like receptors (TLRs), a broad group of recognition molecules in the innate immune system [38,39]. TLR4 (toll-like receptor 4), MD-2 (myeloid differentiation factor 2), and CD14 form the TLR4 complex, which is able to recognizes the lipopolysaccharides (LPS) of Gram-negative bacteria [40,41]. Therefore, we investigated fluctuations in the expression of CD14, besides other surface molecules, such as CD54 (ICAM-1) and CD59 that are involved in cell adhesion and apoptosis, respectively. In fact, ICAM-1 is a cell surface molecule involved in inflammation and preferentially expressed on vascular endothelial cells subjected to pathological factors. As such, ICAM-1 is being explored as a target for intervention against inflammation, immune disorders, cardiovascular diseases, genetic and metabolic syndromes, and cancer, among other conditions [42,43,44,45,46,47,48,49,50,51]. CD59, or Membrane Attack Complex (MAC) Inhibition Factor, is a membrane-bound regulatory protein that inhibits the assembly of the terminal membrane attack complex (C5b-9) of complement, leading to the impairment of pore formation and osmotic lysis. This protein, which is considered a general infection marker in monocytes, can be localized into lipid rafts [52], which act as platforms that enhance cellular signaling [53]. CD59 may also serve as a receptor for the endocytosis of macromolecules into nucleated cells. The endocytosis process is a strategy of particle entry, ubiquitous in non-phagocytic cells [54]. It is not surprising that a variety of microbial pathogens have evolved to exploit aspects of this internalization process as a mechanism to gain entry into host cells [55] and be managed by lysosomes.

Our previous studies demonstrated that *C. jejuni* ATCC 33291 and *C. jejuni* ISS 3 lysates induced apoptosis in HeLa cells. These findings focused on a differential mitochondrial and endo-lysosomal involvement in the cell death pathways induced by lysates from these two different strains [17]. Here, we describe the monocyte response at the subcellular level, detecting mitochondrial and lysosomal network alterations, reactive oxygen species (ROS) increase, and ER stress. Furthermore, we link these functional actions of the lysates to intracellular Ca^2+^ levels: the ER regulates Ca^2+^ homeostasis through the presence of many Ca^2+^ binding proteins that work as buffers by having a low-affinity and large capacity for Ca^2+^ binding. The ER and sarcoplasmic reticulum coordinately regulate cellular Ca^2+^ homeostasis and signaling, together with the mitochondria. Nevertheless, in specific cases, autophagy may induce cell destruction, as a result of a protracted atrophy of the cytoplasm. Finally, we observed that the lysates induced pro-survival and lethal effects as well as alterations in the maturation of acidic organelles and ER stress response. CDT seems not only to be responsible for specific death effects but also to play a role in promoting a pathway for resisting apoptotic stimuli (sublethal effects).

## 2. Results

### 2.1. Morphological Features, Cell Death, and Absolute Count

It has been widely shown how CDT intoxication induces cellular distension and enlargement in target cells [56]. The forward light scatter parameter (FSC) was used as an indicator of cell size in cytometry; these data were taken together with those from microscopy analyses of morphologic appearance.

**Figure 1:**
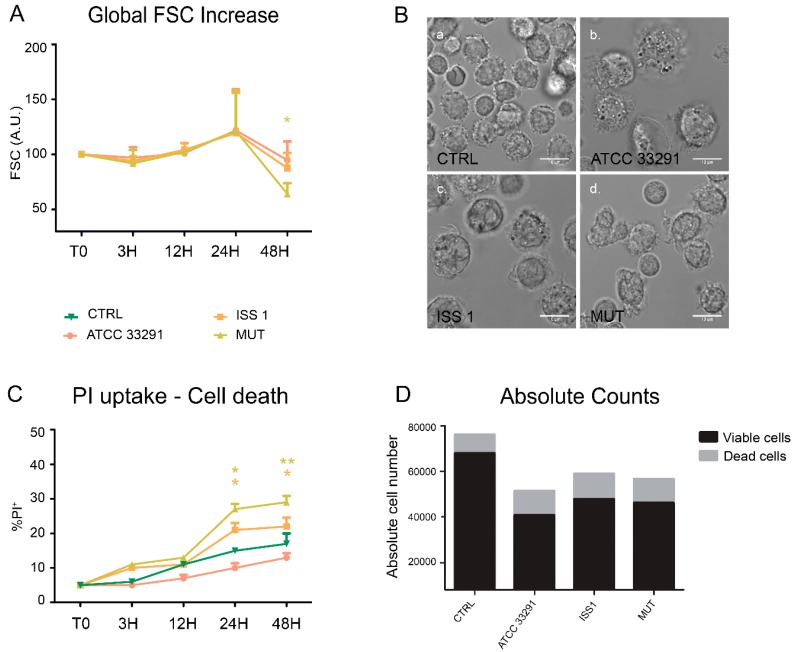
Morphological features and cell death rate. (**A**) Trends of forward light scatter (FSC) values for each treatment during the time course from the start (T0) to 48 h. The FSC values were converted to arbitrary units (A.U.), setting the control (T0) to 100. Each value is expressed as the mean ± SD (results from *n* ≥ 3 independent experiments). Two-way ANOVA with Bonferroni’s multiple comparison test revealed * *p* < 0.05 vs. control (T0); (**B**) Bright-field images of monocytes after 24 h of preincubation with *Campylobacter jejuni* ATCC 33291 lysate (b), *C. jejuni* ISS 1 lysate (c) and *C. jejuni* 11168H *cdtA* mutant lysate (d), compared to the untreated control cells (a). Bars: 10 µm; (**C**) Trends of percentage of propidium iodide (PI)-positive cells for each experimental condition during the time course from T0 to 48 h. Each value is expressed as the mean ± SD (results from *n* ≥ 3 independent experiments); * *p* < 0.05 and ** *p* < 0.01 vs. control (T0); (**D**) Absolute counts of viable and dead cells after 48 h of preincubation with the lysates, compared to untreated control cells. Results from a representative experiment.

We compared the FSC values of untreated control cells with the FSC values of cells that were treated with lysates previously prepared. All treatments induced a moderate increase in the FSC values and cell enlargement after 24 h (Figure 1A). At 48 h, the cells preincubated with lysates from the wild-type strains showed the typical enlargement induced by CDT, whereas the cells preincubated with the *C. jejuni* 11168H *cdtA* mutant lysate revealed a significant reduction in the FSC values, also due to the apoptotic shrinkage that occurred in the latter (see data on propidium iodide positivity).

The PI (propidium iodide) supravital labelling allowed us to detect and discriminate necrotic and apoptotic cells (Figure 1C). The PI results demonstrated that the lysates caused a significant increase in PI-positive cells (apoptotic cells) in all experimental conditions. Of note, from 24 h onwards, both lysates from the wild-type strains acted as death-inducers, but in two different ways. To deeply monitor the phenomenon of cell death, we performed cell count by absolute counting beads in cytometry. The microscopy visualization of this cellular rarefaction and total cell count are shown in Figure 1B, D respectively. The reduction in cell number was particularly evident in monocytes preincubated with the *C. jejuni* ATCC 33291 lysate. 

### 2.2. Mitochondrial and Lysosomal Alterations

**Figure 2:**
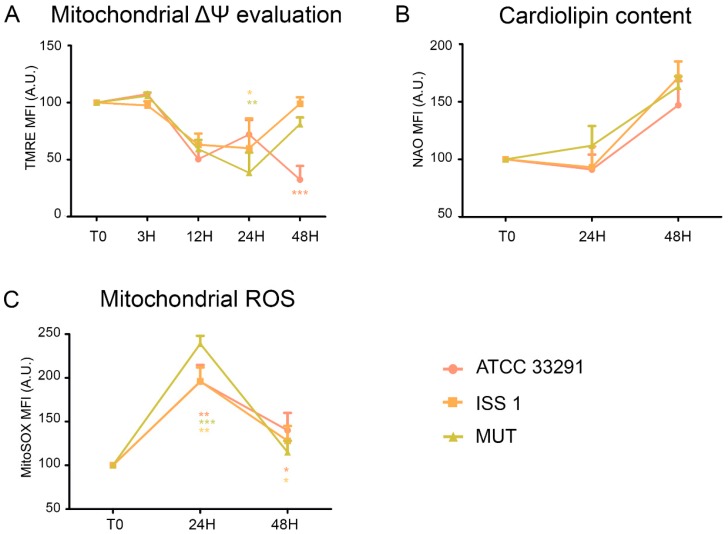
(**A**) Trends of TMRE mean fluorescence intensity (MFI) for each treatment during the time course from T0 to 48 h. Mean values were converted to A.U., setting the control (T0) to 100. Each value is expressed as the mean ± SD (results from *n* ≥ 3 independent experiments). Two-way ANOVA with Bonferroni’s multiple comparison test revealed: * *p* < 0.05, ** *p* < 0.001, and *** *p* < 0.001 vs. control (T0). The trend during the time course was determined to be significant. (**B**) Trends of nonyl acridine orange (NAO) MFI for each experimental condition during the time course from T0 to 48 h. The mean values were converted to A.U., setting the control (T0) to 100. Each value is expressed as the mean ± SD (results from *n* ≥ 3 independent experiments). (**C**) Trends of MitoSOX MFI for each treatment during the time course from T0 to 48 h. The mean values were converted to A.U., setting the control (T0) to 100. Each value is expressed as the mean ± SD (results from *n* ≥ 3 independent experiments) Two-way ANOVA with Bonferroni’s multiple comparison test revealed: * *p* < 0.05, ** *p* < 0.001, and *** *p* < 0.001 vs. control (T0).

Perturbations of mitochondrial transmembrane potential resulted after preincubation with the lysates (Figure 2A). TMRE MFI (mean fluorescence intensity) gradually decreased in monocytes infected with the *C. jejuni* ATCC 33291 lysate during the time course (from 3 to 48 h). At 48 h, these monocytes showed the lowest TMRE value, compared with other experimental conditions, in which a decrease in TMRE MFI was followed by a return to the original, control values (T0). 

Cardiolipin (CL) is an easily oxidizable phospholipid present in the mitochondria. To investigate the mitochondrial status, we also evaluated cardiolipin content by nonyl acridine orange (NAO) staining. As shown in Figure 2B, non-significant variations in cardiolipin content occurred in monocytes preincubated with the lysates. At the same time, by using the MitoSOX staining, we detected a great increase in mitochondrial ROS from 24 h onwards in all experimental conditions, compared to the control (T0) (Figure 2C). 

**Figure 3:**
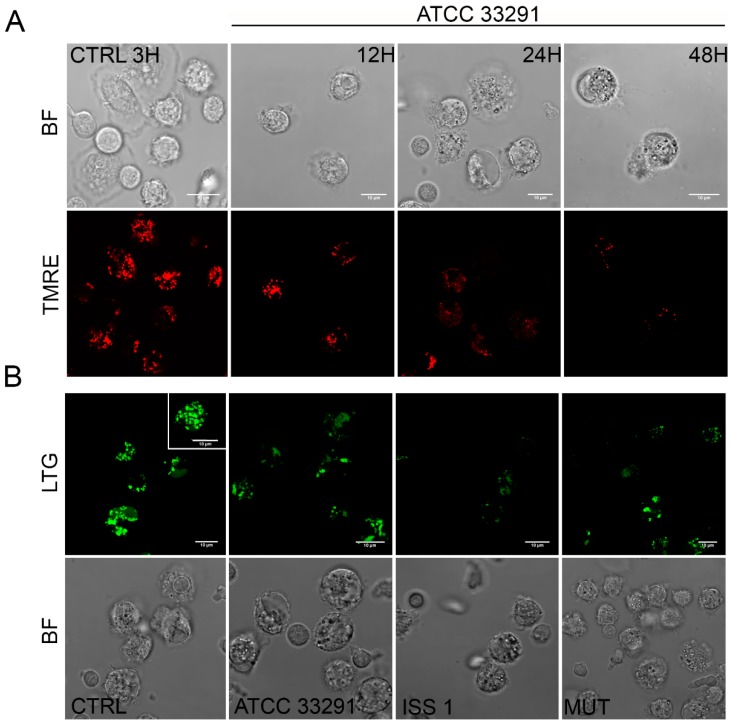
(**A**) Single confocal optical sections of TMRE (mitochondria), with the relative bright-field (BF) images, of CTRL cells and monocytes infected with *C. jejuni* ATCC 33291 lysates for 12, 24, and 48 h. Bars: 10 µm; (**B**) Single confocal optical sections of Lyso Tracker Green (LTG, lysosomes) with the relative BF images for CTRL cells and cells infected with ATCC 33291, ISS 1, and 11168H *cdtA* mutant lysates for 12 h. Bars: 10 µm.

In order to investigate whether the *C. jejuni* lysates altered host lysosome integrity during infection, the cells were stained with Lyso Tracker Green, a lysosomotropic dye that emits fluorescence when it accumulates in acidic compartments such as lysosomes. As shown in Figure 3B, in terms of amounts of LTG-accumulating lysosomes, the *C. jejuni* lysates induced in monocytes a reduction at 12 h followed by a significant increase at 24–48 h, specifically in cells preincubated with the *C. jejuni* ISS 1 lysate. 

These findings are consistent with the PI results that showed that the mortality rate was low at 12 h and pronounced at 24–48 h (Figure 1C). These data also demonstrated that, if the monocytes initially reduced their lysosomal compartments in size, number, and functionality, subsequently they increased them in the late phases of the infection. 

**Figure 4:**
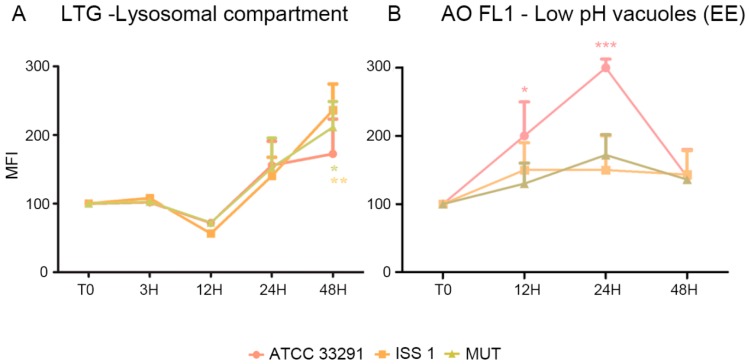
Trends of MFI for each treatment during the time course from T0 to 48 h for LTG (**A**) and AO-FL1 (**B**). the mean values were converted to A.U., setting the control (T0) to 100. Each value is expressed as the mean ± SD (results from *n* ≥ 3 independent experiments). Two-way ANOVA with Bonferroni’s multiple comparison test revealed: (**A**) * *p* < 0.05 and ** *p* < 0.001 vs. control. (**B**) Trends of AO-FL1 MFI for each experimental condition during the time course from T0 to 48 h. The mean values were converted to A.U., setting the control (T0) to 100. Each value is expressed as the mean ± SD (results from *n* ≥ 3 independent experiments). Two-way ANOVA with Bonferroni’s multiple comparison test revealed: * *p* < 0.05 and *** *p* < 0.001 vs. control (T0).

The lysosomes, i.e., the acidic vacuolar organelles (AVOs), were counted by means of flow cytometry as LTG MFI values (Figure 4) and also as AO MFI values for both FL1 and FL3 (emission wavelength from 510 to 650 nm). We do not show data for AO FL3 fluorescence, similar to LTG data, but uniquely for FL1, related to low-PH vacuoles.

Figure 4 shows a comparison between the LTG-traced lysosomal network and AO FL1-traced vacuoles. For ATCC 33291 lysate-treated cells, it is evident an increase in low-PH vacuoles (endosomal compartment) concomitant to an increase in AVOs up to 24 h, whereas, at 48 h, the endosomes decreased, and AVOs reached a plateau level. In contrast, mutant and ISS 1 lysate–treated cells did not particularly increase their content in low-PH vacuoles, whereas the lysosomes peaked.

The early lysosomes (EE) are acidified to pH 6.2 by V-ATPase, allowing the uncoupling of bound ligands from their receptors [57]. By contrast, the late endosomes (LE) and lysosomes are more acidic, with a luminal pH of 5.0–5.5 which is required for the full activation of lysosomal hydrolases. All roads do not lead to the lysosomes in fact; the late endosomal compartment may represent an escape route, due to the possibility to extrude their content. Therefore, we combined these findings together with lysosomal exocytosis data, since lysosomes and LEs are also involved in the secretory pathway.

### 2.3. Surface LAMP-1 Expression: Lysosomal Exocytosis

**Figure 5:**
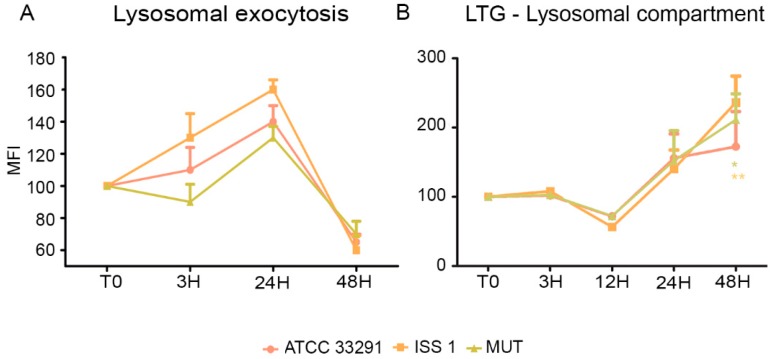
(**A**) Graph showing lysosomal exocytosis for each experimental condition during the time course from T0 to 48 h. The mean values were converted to A.U., setting the control (T0) to 100. Each value is expressed as the mean ± SD (results from *n* ≥ 3 independent experiments); (**B**) Trends of LTG MFI for each treatment during the time course from T0 to 48 h. The mean values were converted to A.U., setting the control (T0) to 100. Each value is expressed as the mean ± SD (results from *n* ≥ 3 independent experiments). Two-way ANOVA with Bonferroni’s multiple comparison test revealed: * *p* < 0.05 and ** *p* < 0.01 vs. control (T0). The trend during the time course was determined to be significant.

Being specialized phagocytic cells, monocytes typically express low levels of surface LAMP-1 (CD107a). In this study, the expression of surface LAMP-1 protein was investigated in infected and uninfected cells. It was observed an increase in LAMP-1 expression principally in monocytes preincubated with the wild-type lysates from T0 to 24 h (Figure 5A). Increasing the exposure time (48 h), a downregulation of LAMP-1 to hypo-physiologic levels occurred. Our data on lysosomal exocytosis suggested that lysosomes, in the first infection stages, may “dump” indigested materials, reducing their accumulation within lysosomes, whereas, at 48 h, a drop in exocytotic activity was registered. These findings were coupled with the LTG results (Figure 5B): in fact, the decrease in lysosomes size and number was not due to exocytosis activation. On the contrary, at 48 h, when LTG fluorescence peaked, LAMP-1 strongly decreased. This process might have an important role in secretion and plasma membrane repair, since the lysosomes play a key role in resealing the membrane to prevent cell death [34].

### 2.4. CD14 and ICAM-1 (CD54) Variations 

Bacterial products have been shown to activate monocytes and to increase CD14 expression. Furthermore, the activation of monocytes increases survival, whereas their deactivation evokes apoptosis (programmed cell death) [58].

This correlation among activation, CD14 expression, and the lifespan of the cells prompted us to investigate the role of CD14 and to monitor potential alterations of this monocytic cell surface marker.

**Figure 6:**
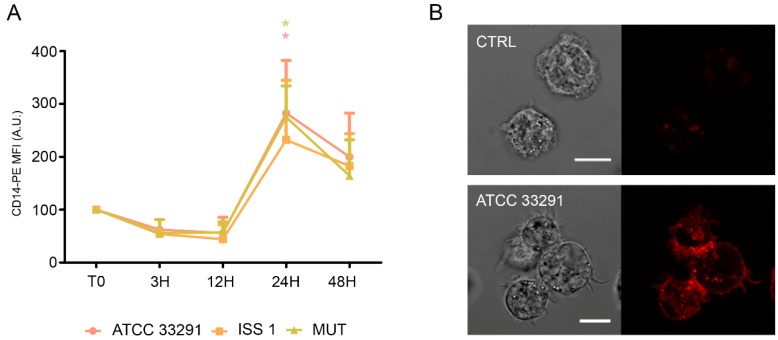
CD14 expression. (**A**) Graph showing CD14 expression in monocytes preincubated with the lysates. The mean values were converted to A.U., setting the control (T0) to 100. Each value is expressed as the mean ± SD (results from *n* ≥ 3 independent experiments). Two-way ANOVA with Bonferroni’s multiple comparison test revealed: * *p* < 0.05 vs. control (T0). The trend during the time course was determined to be significant; (**B**) Confocal images of CD14-PE with the relative BF images from monocyte control cells and monocytes preincubated with the *C. jejuni* ATCC 33291 lysate for 24 h. Bars: 10 µm.

In infected monocytes, an early decrease in CD14 expression (T0–12 h) was followed by an important increase in CD14 expression after 24 h of treatment (Figure 6). 

**Figure 7:**
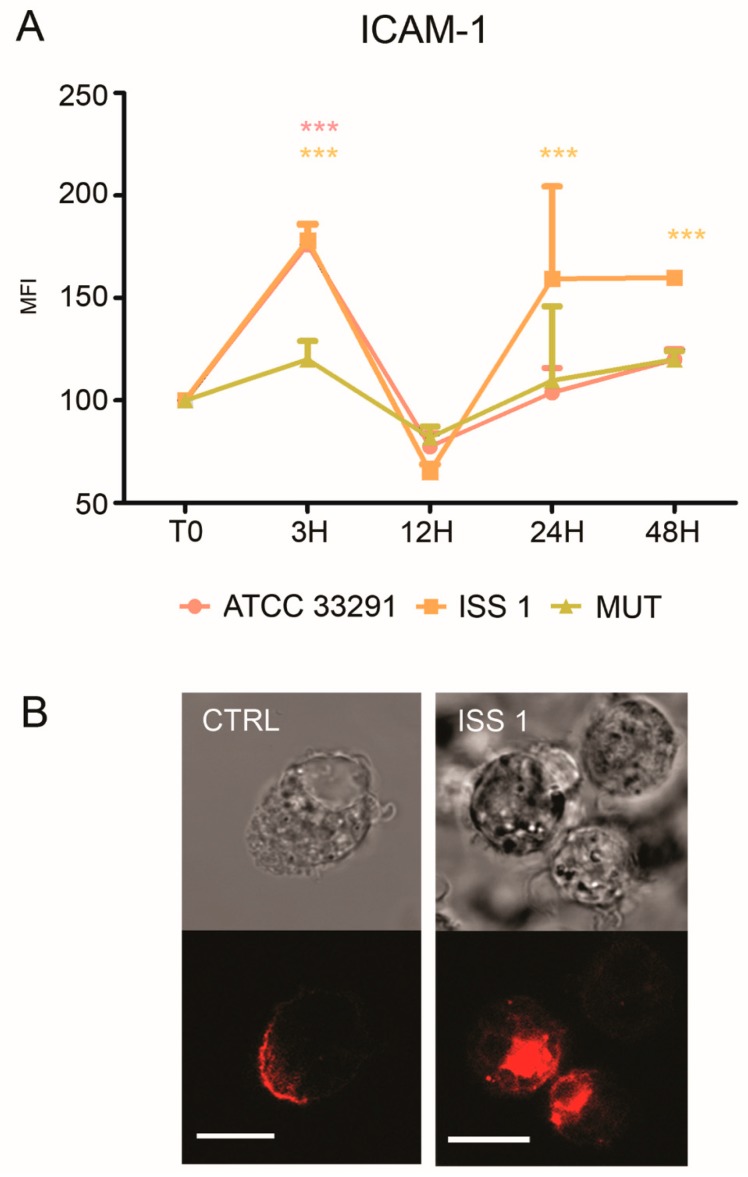
ICAM-1 expression. (**A**) Graph showing ICAM-1 expression. The mean values were converted to A.U., setting the control (T0) to 100. Each value was expressed as the mean ± SD (results from *n* ≥ 3 independent experiments). Two-way ANOVA with Bonferroni’s multiple comparison test revealed: *** *p* < 0.001 vs. control (T0). The trend during the time course was determined to be significant; (**B**) Confocal images of CD54-PE with the relative BF images from monocyte control cells and monocytes preincubated with the ISS 1 lysate after 48 h of treatment. Bars: 10 µm.

Increased ICAM-1 expression induced by various pathogens was shown to mediate cell-to-cell adhesion in inflamed tissues [59,60]. As showed in Figure 7A, an increase in ICAM-1 expression was detectable in monocytes, particularly at 3 and 48 h. Furthermore, confocal pictures revealed a variation in surface ICAM-1 distribution (Figure 7B): a ‘punctuate’ organization with a loss of ‘cap’ distribution occurred, confirming that a reshaping of the plasma membrane arose because of the infection (Figure 7B). 

### 2.5. p53 and Bcl-2 Detection

**Figure 8:**
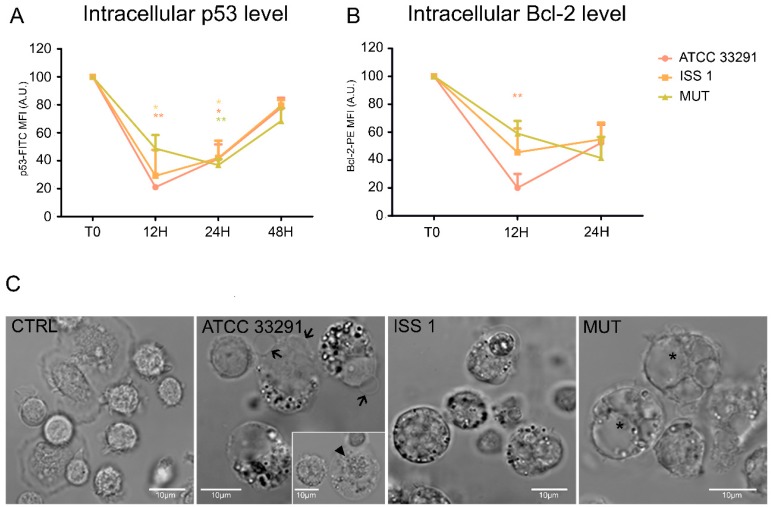
p53 and Bcl-2 protein levels and morphologic features of cell death. (**A**) Graphs showing p53 intracellular content for each experimental condition during the time course from T0 to 48 h. The mean values were converted to A.U., setting the control (T0) to 100. Each value is expressed as the mean ± SD (results from *n* ≥ 3 independent experiments). Two-way ANOVA with Bonferroni’s multiple comparison test revealed: * *p* < 0.05 and ** *p* < 0.01 vs. control (T0); (**B**) Graphs showing Bcl-2 intracellular content for each experimental condition during the time course from T0 to 48 h. The mean values were converted to A.U., setting the control (T0) to 100. Each value is expressed as the mean ± SD (results from *n* ≥ 3 independent experiments). Two-way ANOVA with Bonferroni’s multiple comparison test revealed: ** *p* < 0.01 vs. control (T0); (**C**) BF images of monocytes after 24 h of preincubation with *C. jejuni* ATCC 33291, ISS 1, and 11168H *cdtA* mutant lysates, compared to untreated control cells (CTRL). In infected cells, it is possible to appreciate apoptotic blebs (black arrows), condensed nuclei (arrowhead), cytoplasmic vacuoles (asterisks), and a diffuse granulation (in particular in ATCC 33291 and ISS 1). Bars: 10 µm.

Our results revealed a decrease in p53 content in monocytes preincubated with the wild-type lysates for 12 h. This status remained stable for 24 h, and then p53 intracellular content increased, reaching the initial physiological levels (Figure 8A). These fluctuations reflected the balance between growth arrest and apoptosis mechanisms [26]. Apoptosis was accompanied by morphological changes, including nucleus condensation, apoptotic blebs formation, and appearance of cytoplasmic vacuoles and granulation (Figure 8C).

The apoptotic threshold of a cell was determined by a ratio of pro-apoptotic and anti-apoptotic signals, and apoptosis occurred when the pro-apoptotic signals outweighed the counteracting anti-apoptotic factors [61].

Anti-apoptotic Bcl-2 family proteins are known to influence the apoptotic threshold. Our findings revealed the following scenario for Bcl-2 (Figure 8B): from T0 to 12 h, a drop in Bcl-2 intracellular levels was observed, in particular in monocytes preincubated with the *C. jejuni* ATCC 33291 lysate; at 24 h, Bcl-2 values in all experimental conditions were very similar.

### 2.6. ER Stress and Modulation of Intracellular Ca^2+^ Levels

**Figure 9:**
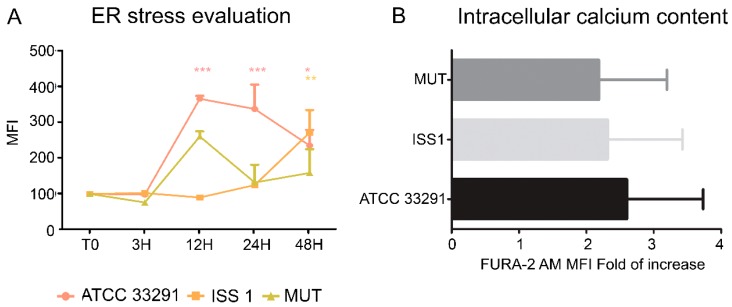
(**A**) Graph showing ER-Tracker Green MFI for each experimental condition during the time course from T0 to 48 h. The mean values were converted to A.U., setting the control (T0) to 100. Each value is expressed as the mean ± SD (results from *n* ≥ 3 independent experiments). Two-way ANOVA with Bonferroni’s multiple comparison test revealed: * *p* < 0.05, ** *p* < 0.01, and *** *p* < 0.001 vs. control (T0); (**B**) Fold of increase in intracellular Ca^2+^ content, measured by means of FURA-2 AM, referred to control samples after 48 h of preincubation. Each value is expressed as a fold of increase ± SD (results from *n* ≥ 3 independent experiments).

ER stress might be both a trigger and consequence of chronic inflammation. Monocytes were stained with ER-Tracker Green and Fura-2 AM in order to investigate ER stress and intracellular Ca^2+^ level perturbations. ER-Tracker Green dye is a cell-permeant, live-cell stain that is highly selective for the ER. It stains ER membranes and it is known to be an ER stress marker in both cytometry and microscopy [62]. Our data showed that ER stress occurred after 12–24 h of treatment (Figure 9A), especially in monocytes infected with the *C. jejuni* ATCC 33291 lysate. Moreover, the lysate from the *cdtA* mutant strain caused more ER modifications then the that from the ISS 1 wild-type strain; we supposed that ER stress was not an effect associated with CDT, but it was due to other *C. jejuni* virulence factors. In addition, the FURA-2 AM results showed an increase in intracellular Ca^2+^ content in monocytes preincubated for 48 h with the *C. jejuni* ATCC 33291 lysate compared with other experimental conditions (data not statistically significant) (Figure 9B). 

### 2.7. Surface CD59 Expression

**Figure 10:**
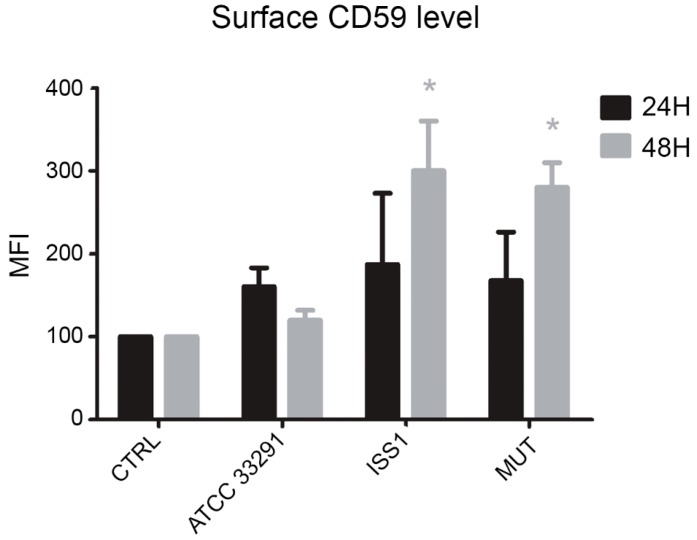
Bar graph of CD59 expression for each experimental condition after 24 and 48 h of treatment. The mean values were converted to A.U., setting the control (T0) to 100. Each value is expressed as the mean ± SD (results from *n* ≥ 3 independent experiments) Two-way ANOVA with Bonferroni’s multiple comparison test revealed: * *p* < 0.05 vs. control (T0).

CD59 expression was studied in monocytes in order to investigate if the MAC complex was involved in the cell response triggered by *C. jejuni* lysates. It was found that, from 24 to 48 h, in infected monocytes, upregulation of CD59 surface expression occurred: it was particularly evident in cells preincubated with the *C. jejuni* ISS 1 lysate, followed by monocytes preincubated with the mutant strain lysate (Figure 10). This condition, being CD59 a critical complement regulator in protecting human cells from MAC formation and MAC-induced phenomena, seems to predispose the monocytes to survive. In fact, it is known that CD59 overexpression may assist malignant cells to escape immunologic surveillance and complement-mediated cytolysis [61].

## 3. Discussion

Our results highlight that cellular distension, typically induced by CDT, was mainly appreciable in monocytes preincubated with lysates from the wild type strains, particularly after 48 h. During the time course, cell death increased, qualifying the *C. jejuni* ATCC 33291 lysate as the best cell death-inducer, followed by the *C. jejuni* ISS 1 lysate. We can state that a reduction in PI-positive cells at 48 h in monocytes treated with the *C. jejuni* ATCC 33291 strain had to be coupled with other cell death parameters (i.e., absolute cell counts).

In order to evaluate mitochondrial mass and morphology, as well as the involvement of mitochondria in the apoptotic pathway, cells were stained with TMRE and NAO, two mitochondria-specific fluorochromes. The collapse of the mitochondrial transmembrane potential leads to the opening of the mitochondrial permeability transition pores, the release of cytochrome *c* into the cytosol, and the activation of the apoptotic cascade. Depending on the specific apoptotic stage in which cells are, the mitochondrial transmembrane potential can increase or decrease: more polarized mitochondria (i.e., hyperpolarized, where the interior is more negative) will accumulate more cationic dye, whereas depolarized mitochondria (where the interior is less negative) will accumulate less dye because of an extremely collapsed mitochondrial status [63]. The *C. jejuni* strain that induced the most relevant mitochondrial alterations was the ATCC 33291, confirming that this strain favors an intrinsic apoptotic pathway. In addition, an increase in the content of reactive oxygen species occurred in infected monocytes, probably in a CDT-independent manner, as demonstrated by the MitoSOX results, which are consistent with the TMRE results. We found mitochondria and lysosomes differently targeted by the different strains in monocytes. Indeed, whereas the *C. jejuni* ATCC 33291 wild-type strain showed to induce an intrinsic apoptotic pathway, characterized by the induction of mitochondria alterations, the *C. jejuni* ISS 1 wild-type strain mostly induced lysosomal alterations. Furthermore, from 3 to 12 h, the trend was the same for all experimental conditions: a significant decrease in LTG fluorescence indicated that a neutralization of the host acidic compartments occurred in the infected cells in response to *C. jejuni* virulence factors contained within the lysates. At 48 h, the host acidic compartments/lysosomes started to respond differently to the different lysates, and, at the same time, lysosome/LE exocytosis was strongly inhibited, and a specific behavior of Early Endosomes (low-PH vacuoles) was observed, suggesting alterations in endosomal trafficking. 

A previous publication [64] studying fluorescent probes as acidic vesicle tracers for confocal fluorescence imaging and quantitative analysis, put in light that the amount of AO accumulated in each vesicle and the corresponding color of fluorescence were most likely a direct consequence of the intravesicular low pH which is responsible for the protonation of the dye molecules and its subsequent trapping. It was observed that, quite frequently, two adjacent vesicles differed in color, one exhibiting green, and the other exhibiting red AO luminescence, indicating that such a pair may have been an endosome and a lysosome at the stage immediately prior to fusion [64], while this was not true for cells treated with mutant and ISS 1 lysates, since lysosome–endosome fusion is required for endosome maturation and killing of ingested microorganisms but not self-particles. We can assume that, for ATCC 33291 administration, this step was regularly performed by the cells, whilst in ISS 1 treated-cells, we observed a stabilization of the lysosomal membrane (the highest MFI values), contemporary to a partial inhibition of the mitochondrial membrane signaling pathway. It is conceivable that cells treated with the lysates from the other two strains suffered from an increase or promotion of endosome maturation–acidification (particularly evident for ISS 1) that led to lysosome and LE increase.

In fact, it is known that, in monocytes and macrophages, phagosome/endosome maturation proceeds quickly, whereas in our cellular model this did not happen. Differences between the various applied *C. jejuni* strains may be due to various factors that have direct or indirect impacts on phagosome/endosome maturation, such as pathogen-associated molecular patterns (PAMPs), damage-associated molecular patterns (DAMPs), or cytokines. Of note, the general effect induced by the *C. jejuni* ATCC 33291 lysate converged on the apoptotic intrinsic pathway, whereas this was not verified for the *C. jejuni* ISS 1 lysate. We can assume that, in order to delay phagosome maturation and enhance antigen presentation, *C. jejuni* ATCC 33291 and ISS 1 strains used CDT toxin and LPS sinergically [65,66], whereas the *C. jejuni* cdtA mutant strain exclusively used LPS stimulation.

Changes in lysosomal acidic compartments and in ER and p53 expression occurred together from T0 to 24 h. The p53 tumor suppressor responds to certain cellular stresses by inducing transcriptional programs that can lead to growth arrest or apoptosis. p53-dependent growth arrest and apoptosis are usually caused by stimuli that trigger DNA damage [67]. In this investigation, the lysates from the wild-type strains induced an important decrease in p53 intracellular content from T0 to 12 h; this effect was not shown in cells preincubated with the lysate from the mutant strain. This finding underlines that this specific effect is mainly due to CDT. Different authors have demonstrated that the degradation of p53 prolongs pathogen survival [68]. However, p53 levels increased after 48 h. This increase might be also associated with data about the lysosomal compartments and lysosomal exocytosis at the same time point. Our findings also revealed a strong decrease in Bcl-2 content, specifically after 12 h, in monocytes treated with the ATCC 33291 lysate: this pro-apoptotic response is consistent with the deep mitochondria alterations induced by this strain that have been already described.

The lysates from all strains induced ER stress in monocytes compared to the untreated control cells; of note, an important stress was induced by the *C. jejuni* ATCC 33291 strain. For the reason that the mutant lysate caused more ER alterations than the ISS 1 lysate, we suppose that ER stress was not associated with CDT but to other *C. jejuni* virulence factors. The ER data are consistent with the increase in cytosolic Ca^2+^ content induced by the lysates. 

CD59 is an 18 kDa cell surface protein that inhibits the formation of the MAC, the pore forming toxin of the complement system that cells activate in order to kill pathogens. The marked increase of CD59 expression observed in monocytes treated with the lysates could promote the pro-inflammatory and non-lytic role of MAC, resulting in the protection of the bacterium and virulence factors from complement attack. CD59 was revealed to be a general infection marker in monocytes. The maximum increase of CD59 was registered at 48 h for ISS 1 and 11168H *cdtA* mutant lysates, whereas the cells treated with the *C. jejuni* ATCC 33291 lysate showed a reduction at this time point: this is consistent this is consistent with the activation, above demonstrated, of the apoptotic process. Upregulation of CD59 in monocytes preincubated with the mutant strain suggested that CDT was not implicated in the processes described above. Although the monocytes express basal lysosomal exocytosis processes because of their phagocytic nature, LAMP-1upregulation was caused by the lysates used in our investigations. However, our findings suggest that virulence factors contained in the lysates are able to activate lysosomal exocytosis in monocytes in a CDT-dependent manner. CD14 involvement in the host defence against viral and bacterial infections has been investigated in several experimental models. It was reported that CD14 is upregulated by bacterial LPS and contributes to CD14-mediated phagocytosis of Gram-negative bacteria [69]. In this work, we did not find particular differences in CD14 expression between cells preincubated with the wild-type lysates and those incubated with the mutant strain lysate; on the contrary, variations between uninfected and infected cells and variations in CD14 expression during the time course were statistically significative. ICAM-1 mobility is of general importance for immune responses that require firm adhesion [70]. Adhesion molecules, such as ICAM-1, direct immune cells to the inflammation site by the process of rolling, activation, adhesion, and transmigration [71]. Altered ICAM-1 mobility perturbs intercellular adhesion. In this work, ICAM-1 expression showed marked fluctuations from T0 to 48 h, peaking after 3 h of treatment and dropping at 12 h, with a subsequent increase at 48 h. Being this behavior mainly evident in cells preincubated with the lysates from the wild-type strains, it is possible to affirm that CDT is involved in these modifications. To better understand the role of the cdtA subunit, in the future, it may be interesting to make the complement of the *cdtA* mutant strain and to investigate if modifications induced by the lack of the cdtA subunit could be restored by a complementation mutant.

## 4. Experimental Section

### 4.1. Ethics Statement

PBMCs (peripheral blood mononuclear cells) were isolated from buffy coats of anonymized donors obtained from the Transfusion Centre of Urbino Hospital. No specific approval from an institutional review board was required for the use of the buffy coats for the following reasons: (1) no personal patient information was made available; (2) buffy coats could not be used for the treatment of patients and were waste products for the blood transfusion center, and (3) the blood donors were verbally informed that parts of the donation that could not be used for patient treatment might be used for scientific research.

### 4.2. Growth Conditions

The *C. jejuni* strains were revived from −80 glycerol stocks by pipetting onto blood agar plates containing autoclaved Columbia agar base (Oxoid, Basingstoke, UK) supplemented with 7% (*v*/*v*) horse blood (Oxoid, Basingstoke, UK) and *Campylobacter* Selective Supplement (Oxoid, Basingstoke, UK). The strains were routinely sub-cultured every 3–4 days up to a maximum of 10 passages. The *C. jejuni* cultures were grown in pre-equilibrated Brucella broth (Oxoid, Basingstoke, UK) in 50 mL bottles with shaking at 120 rpm on a 3005 analogue orbital shaker (GFL, Burgwedel, Germany) in microaerophilic conditions.

### 4.3. Cell Lines and Monocytes Isolation

PBMCs were isolated from freshly collected buffy coat preparations of whole human blood from healthy donors. To harvest the monocytes from the peripheral blood, the buffy coats were subjected to a double density gradient centrifugation, as described below [72]. A volume of 30–35 mL of buffy coat was layered on top of the Ficoll-Paque^TM^ Plus solution (GE Healthcare, Little Chalfont, UK) into a 50 mL centrifuge tube for the first density gradient. The sample was centrifuged at 400× *g* without brake for 30 min at room temperature (RT), and the white ring of PBMCs, located between the two phases, was collected with a serological pipette and was transferred to a 50 mL centrifuge tube. The tube was filled with PBS–EDTA (1 mM) up to 40 mL in total, and centrifuged at 300× *g* for 10 min without brake at RT. The resultant supernatant was aspirated, and the pellets were washed with 40 mL PBS–EDTA (1 mM). For each donor, the resultant pellets, containing PBMCs, were polled in 20 mL RPMI-1640 without phenol red + 10% FCS. For the second density gradient, an iso-osmotic Percoll solution (density: 1.131 g/mL) was prepared, as described in a protocol [72]. For each donor, 25 mL of the prepared Percoll solution was transferred to a 50 mL tube, and the PBMC solution previously prepared was layered on the top of the Percoll solution. The tubes were centrifuged at 550× *g* without brake for 30 min at RT. For each gradient, the white ring containing monocytes, located between the two phases, was collected with a serological pipette and transferred to a 50 mL centrifuge tube. The monocytes were washed with PBS–EDTA (1 mM) by centrifugation at 400× *g* for 10 min without brake at RT. The supernatant was aspirated, and pellets were resuspended in RPMI 1640 medium supplemented with 10% FBS, 2 mM l-glutamine, 100 U/mL penicillin, and 100 mg/mL streptomycin, and incubated in 5% CO_2_ at 37 °C in polylysine-coated six-well tissue culture plates. After 24 h, the non-adherent cells were removed by gentle washing, and the adherent cells left in culture were used for the subsequent assays. 

### 4.4. C. jejuni Cell Lysate Preparation

*C. jejuni* ATCC 33291, *C. jejuni* ISS 1, and *C. jejuni* 11168H *cdtA* mutant strains were grown in 50 mL Brucella broth at 37 °C in a shaking incubator under microaerophilic condition for 48 h. The bacterial suspensions were adjusted spectrophotometrically to approximately 10^8^ bacteria/mL and centrifuged at 4000 rpm for 10 min. The pellets were resuspended in 20 mL of Dulbecco’s modified eagle medium (D-MEM) (Sigma-Aldrich, St. Louis, MO, USA) and lysed by sonication (2 × 30 s bursts with 30 s intervals between each burst) by using a sonicator (Sonifier 450, Branson, MO, USA). The cell debris and unlysed bacterial cells were then removed by centrifugation at 4000 rpm for 10 min. Aliquots of each lysate were sterilized by 0.22 µm membrane filtration (Millipore, Milan, Italy) and stored at −20 °C before use.

### 4.5. Pretreatment of the Monocytes with C. jejuni Lysates

The monocytes were incubated for 6, 12, 24, 48, and 72 h with 2 mL of medium enriched with *C. jejuni* cell lysates (1:100 dilution) from ATCC 33291, ISS 1, and 11168H *cdtA* mutant strains previously prepared. The treated cells were analyzed by means of flow cytometry and confocal microscopy to evaluate different cellular parameters. For the negative control, the cells were incubated with medium only.

### 4.6. Morphological Feature Evaluation

To evaluate changes in cell morphology and size, both cytometry and confocal microscopy were used. In flow cytometry, populations that differed in size and morphology were distinguished on the basis of their physical characteristics: forward scatter (FSC, cell size) and side scatter (SSC, cell granularity). Although a quantitative analysis was carried out in flow cytometry, a qualitative analysis was performed in confocal microscopy. 

### 4.7. Flow Cytometry and Confocal Microscopy Stainings

#### 4.7.1. Flow Cytometric Detection of Cell Death and Flow Cytometric Absolute Count

Cell death features were evaluated using supravital Propidium Iodide (PI) (Sigma-Aldrich, St. Louis, MO, USA) that is capable of binding and labelling DNA. Non-permeabilized cells were incubated 30 min in the dark with 50 µg/mL PI. The cells were washed with PBS and then analyzed by flow cytometry. Apoptotic and necrotic cells were detected as PI^dim^ and PI^bright^ clusters, respectively. 

To investigate programmed cell death features (early and late apoptotic as well as necrotic cells) a double staining with FITC-conjugated Annexin V–PI (AnxV–PI) was performed. AnxV allows detecting phosphatidylserines exposed on the outer cell membrane following caspase activation. AnxV–PI staining was carried out in according to the manufacturer‘s instructions (Immunostep, Salamanca, Spain).

Absolute cell counting was performed by using Dako CytoCount^TM^ beads (Thermo Fisher Scientific, Waltham, MA, USA). A total of 200 µL of sample was carefully dispensed at the bottom of a tube, and 50 µL beads were added. The samples were analyzed by using a FACSCanto cytometer (Becton Dickinson, BD, Franklin Lakes, NJ, USA) within 60 min. Approximately 20,000 cell events were collected. Setup and calibration procedures were optimized for the absolute counting protocols [73].

#### 4.7.2. Determination of Mitochondrial Potential (ΔΨm), Mitochondrial Mass, and Mitochondrial Reactive Oxygen Species (ROS)

Mitochondrial features were investigated by TMRE, NAO, and MitoSOX stainings. Tetramethylrhodamine ethyl ester perchlorate (TMRE) (Sigma-Aldrich, St. Louis, MO, USA) is a ΔΨm-specific stain able to selectively enter the mitochondria depending on ΔΨm, producing red fluorescence. TMRE 40 nM was added to the sample 15 min before the acquisition time. The samples were analyzed by confocal microscopy and flow cytometry using the appropriate fluorescence channel [17].

The cardiolipin-sensitive probe nonyl acridine orange (NAO) (Sigma-Aldrich, St. Louis, MO, USA) is able to monitor changes in mitochondrial lipids [74,75].

In the present study, this probe was used to measure the mitochondrial mass and number independently of the mitochondrial membrane potential (ΔΨ). After a 15 min incubation at 37 °C in the dark with 100 nM NAO, the samples were analyzed by flow cytometry using the appropriate fluorescence channels.

MitoSOX Red (Thermo Fisher Scientific, Waltham, MA, USA) is a fluorigenic dye specifically targeted to mitochondria in live cells. Oxidation of this probe by superoxide that is contained in the mitochondria produces a red fluorescence. MitoSOX, 5 µM, was added to the samples 10 min before the time of acquisition. The samples were analyzed by flow cytometry using the appropriate fluorescence channel [75].

#### 4.7.3. Assessment of Lysosomal Involvement

To label and trace lysosomes in monocytes, the acidotropic dye LysoTracker Green (LTG) (Thermo Fisher Scientific, Waltham, MA, USA) was used. LysoTracker^®^ probes typically concentrate in spherical acidic organelles within live cells, allowing the evaluation of their size and volume [76]. The cells were cultured at 37 °C and resuspended in pre-warmed (37 °C) medium containing 100 nM LysoTracker (diluted in RPMI). After 45 min of incubation, green lysosomal fluorescence was detected by flow cytometry and confocal microscopy. Furthermore, we detected lysosomal involvement using the pH-sensitive dye acridine orange (AO; Sigma-Aldrich, St. Louis, MO, USA), widely used to detect acidic vesicular organelle formation [17]. AO is a cell-permeable fluorescent dye that, at its highest concentrations, stains the DNA red and the cytoplasm bright green. It can also enter acidic compartments, such as lysosomes and autolysosomes, where it becomes protonated and sequestered. At its lowest concentrations, in an acid environment, AO emits red fluorescence with an intensity proportional to the degree of acidity and/or to the acidic compartment volume [77]. Therefore, acidic vesicular organelle formation in AO-stained cells can be measured by flow cytometry. Following treatment with the *C. jejuni* lysates, the cells were washed and resuspended in 0.5 mL RPMI, then stained with acridine orange, 75 ng/mL, for 15 min at 37 °C. Red lysosomal and green cytoplasmic fluorescence of 10,000 cells per sample were acquired by flow cytometry using the FL3 and FL1 channels, respectively.

#### 4.7.4. ER Stress Evaluation

ER-Tracker Green (Thermo Fisher Scientific, Waltham, MA, USA) is a live-cell stain highly selective for the ER. This stain consists of the green fluorescent BODIPY^®^ FL dye and glibenclamide that binds to the sulphonylurea receptors of ATP-sensitive K^+^ channels which are prominent on the ER and have a critical role in ER luminal homeostasis. Indeed, ER K^+^ channels are involved in functions such as protein folding, apoptosis, and calcium homeostasis [78,79]. PBMC were incubated with 100 nM ER-Tracker Green for 30 min at 37 °C and subjected to flow cytometry and confocal analyses.

#### 4.7.5. Intracellular Ca^2+^ Levels Detection

To measure the intracellular Ca^2+^ levels, FURA-2 AM (Thermo Fisher Scientific, Waltham, MA, USA) was used, which is known as an intracellular Ca^2+^ indicator, ratiometric, and UV light-excitable. After treatment with the lysates, the cells were stained with FURA-2 AM, 2 µM, for 15 min at 37 °C. After incubation, the samples were analyzed by flow cytometry, using the appropriate fluorescence channel.

#### 4.7.6. Surface Receptor Expressions Evaluation

To evaluate CD54 (ICAM-1), CD59, and CD14 expression, fluorochrome-conjugated monoclonal antibodies were added to 50 µL of cell pellets. The mouse monoclonal anti-human antibodies anti-CD54-PE conjugated (clone D3.6) (Exalpha Biologicals, Shirley, MA, USA), anti-CD59-Pe-Cy5 conjugated (BD, Franklin Lakes, NJ, USA), and anti-CD14-PE conjugated (clone M5E2) (BD, Franklin Lakes, NJ, USA) were added after dilution according to the manufacturer’s instructions. After 15 min of incubation at RT, the samples were analyzed by flow cytometry and/or confocal microscopy. 

#### 4.7.7. Intracellular Detection of Bcl-2 and p53 Antigens

The monocytes were washed in PBS for 10 min at RT, resuspended in 300 µL of formaldehyde 3.7%, and incubated at 4 °C for 15 min. A volume of 2 mL permeabilization–washing buffer was added, and the cells were centrifuged at 1200 rpm for 10 min. The pellets were resuspended in 300 µL of Cytoperm reagent [80]. Monoclonal anti-human antibodies anti-Bcl-2 PE-conjugated (clone N46-467) (BD, Franklin Lakes, NJ, USA) and anti-p53 FITC-conjugated (clone D0-8) (BioLegend, London, UK) were added to the samples at concentrations according to the manufacturer’s instructions. The cells were incubated at 4 °C for 30 min before being processed by flow cytometry and/or confocal microscopy.

#### 4.7.8. LAMP-1 Surface Expression (Lysosomal Exocytosis Assay)

Cell surface CD107a (LAMP-1), which is found on lysosomes and intracellular lytic granules, was measured. LAMP-1 surface expression was used as a marker of lysosomal exocytosis [81]. CD107a-PeCy5 antibody (clone H4A3, BioLegend, London, UK) was added to 50 μL of cellular suspension at the concentration indicated in the manufacturer’s instructions. The cells were incubated for 1 h at RT and analyzed by flow cytometry. To evaluate lysosomal exocytosis, particularly at 48 h, LAMP-1 values are associated with the cytosolic Ca^2+^ content [82].

#### 4.7.9. Cytometric Investigations

The cytometric experiments were carried out with a FACSCanto II flow cytometer (BD, Franklin Lakes, NJ, USA) equipped with an argon laser (Blue, Ex 488 nm), a helium–neon laser (Red, Ex 633 nm), and a solid-state diode laser (Violet, Ex 405 nm). The analyses were performed with FACSDiva^TM^ software (version 4.1.1, BD, Franklin Lakes, NJ, USA); approximately 10,000 cell events were acquired for each sample.

#### 4.7.10. Confocal Microscopy Analyses

Confocal microscopy analyses were performed by a Leica TCS SP5 II confocal microscope (Leica Microsystem, Wetzlar, Germany) with 488, 543, and 633 nm illuminations and oil-immersed objectives. For confocal live imaging, the cells were grown on MatTek glass bottom chambers (MatTek Corporation, Bratislava, Slovak Republic). The images were further processed and analyzed in ImageJ software (version 1.34e, NIH, Bethesda, MD, USA).

### 4.8. Statistical Analyses

Data are shown as mean ± standard deviation (SD) of at least three independent experiments. Analyses of variance (ANOVA) approaches were used to compare values among more than two different experimental groups for data that met the normality assumption. One-way ANOVA or two-way ANOVA were followed by a Bonferroni post-hoc test. The means of two groups were compared by using the *t* test; *p* values less than 0.05 were considered statistically significant. All statistical analyses were done using GraphPad Prism 5.0 (GraphPad Software, La Jolla, CA, USA).
